# Challenges in tackling tuberculosis on the Thai-Myanmar border: findings from a qualitative study with health professionals

**DOI:** 10.1186/s12913-015-1129-0

**Published:** 2015-10-09

**Authors:** Aiko Kaji, Sein Sein Thi, Terrence Smith, Prakaykaew Charunwatthana, Francois H. Nosten

**Affiliations:** Department of Global Community Health and Behavioral Sciences, School of Public Health and Tropical Medicine, Tulane University, New Orleans, USA; Shoklo Malaria Research Unit, Mahidol-Oxford Tropical Medicine Research Unit, Faculty of Tropical Medicine, Mahidol University, Mae Sot, Thailand; California Analytica, Orange, USA; Mahidol-Oxford Tropical Medicine Research Unit, Faculty of Tropical Medicine, Mahidol University, Bangkok, Thailand; Department of Clinical Tropical Medicine, Faculty of Tropical Medicine, Mahidol University, Bangkok, Thailand; Nuffield Department of Medicine, Centre for Tropical Medicine, University of Oxford, Oxford, UK

**Keywords:** Tuberculosis, Migrants, Organizations, Collaboration

## Abstract

**Background:**

Myanmar and Thailand belong to the top 22 high burden countries for tuberculosis (TB). Health care organizations play an essential role in addressing TB control in the two bridging border jurisdictions, Tak province, Thailand and Myawaddy district, Kayin state, Myanmar. However, health professionals face difficulties in TB control effort due to the nature of fluid population movements, resource constraints and ambiguous mechanisms to implement collaboration along the border. The purpose of this study is to identify the challenges to TB control among Myanmar migrants faced by stakeholders, focusing on the area of collaboration and interaction along the border.

**Method:**

The study conducted in-depth interviews with health policy makers and health care providers responsible for developing and implementing policies and TB programs in Tak province, Thailand and Myawaddy district, Kayin state, Myanmar. The participants included members of government organizations, United Nations agencies, community based organizations, and international NGO. One or two key stakeholders from each organization were approached to participate in the study. We gathered baseline information to identify TB policies and programs available on websites, brochures, and publications. Observations including field notes were made on site. The data transcriptions were coded for qualitative data analysis. Coding also developed categories that led to key themes.

**Results:**

A total of 31 respondents (18 in Thailand and 13 in Myanmar) participated in the study. The main theme reported by participants was challenges in limited corroboration and coordination among stakeholders. Unstructured information sharing and lack of communication hindered the stakeholders from engaging in TB control. The respondents stressed that referral mechanisms across the border need to be strengthened. Other challenges were associated with increasing loss to follow up and subsequent MDR cases, constraints of service delivery, shortage of human resources, limited staff capacities within organizations and poor socioeconomic status of patients.

**Conclusions:**

Health professionals face many challenges in effectively addressing TB control. Addressing the insufficient coordination and collaboration by strengthening bi-national collaborative mechanisms among health care organizations is an essential step in reducing the burden of disease. Additional support and resources from governmental and non-governmental agencies will be required to address the challenges.

## Background

Globally, tuberculosis (TB) remains a major health problem [1]. Despite the availability of TB treatment, in 2013 worldwide there were an estimated 9.0 million new cases and 1.5 million deaths [1]. Nearly 40 % of the incidences were in south-eastern Asia [2]. TB frequently occurs in vulnerable populations such as homeless, refugees, and migrants [3, 4]. Mobile populations have accelerated the spread of infectious diseases including TB [5]. Myanmar and Thailand belong to the top 22 high burden countries for TB in 2013 [1]. Among 1.3 million international migrants who held work permits in Thailand in 2009, 82 % were from Myanmar [6]. In addition, approximately 116,000 displaced persons from Myanmar are living in nine temporary shelters along the Thai-Myanmar border [7]. A study in 2007 reported the prevalence of diagnosed TB patients in Tak province, Thailand was 109 per 100,000 Thai citizens, among Myanmar refugees 340 per 100, 000 and among Myanmar migrants 150 per 100,000 [8]. The study also showed that multidrug-resistant TB (MDR-TB) was diagnosed in 27 (1.6 %) out of 1662 TB patients tested, of whom 19 (70 %) were non-Thais [8]. These statistics highlight the fact that not only TB is more prevalent in the refugee and migrant populations but also that MDR-TB is a significant issue among Myanmar refugees and migrants living in Thailand.

The case notification rate for new smear positive TB cases in Myawaddy township, Myanmar which is located opposite Tak province, was higher than the Myanmar national average. The rate for Myawaddy was 178 per 100,000 whereas the country case notification rate was 88 per 100, 000 in 2012 [9]. These epidemiological findings in Tak and Myawaddy demonstrate that the gateway for migrants is also a pathway for the transmission of TB.

It is crucial for health care providers to identify TB patients and support treatment adherence for TB patients who have the potential to move to another place and to facilitate continuation of their treatment. Health care organizations play a key role in addressing loss to follow up and to improve access to TB care for casual cross-border migrants [9]. Yet cross-border migrants have difficulties in receiving the Directly Observed Treatment Short-course (DOTS) and completing 6 month-treatment at the same health care organization due to a high level of population mobility, poor access to services and insecurity [10, 11]. In a recent study, Médecins Sans Frontières reported that death and default rates for migrant workers were higher than the rates for refugees [12]. The high defaulter rate poses a public health threat.

To date, there is no exploratory study on challenges to TB control along the Thai-Myanmar border from the perspective of stakeholders. Following a qualitative approach, the purpose of this study is to identify challenges to TB control, focusing on the areas of interaction and collaboration among the governmental and non-governmental agencies working in TB control for the two bridging border towns, Tak province, Thailand and Myawaddy district, Kayin state, Myanmar.

## Methods

### Study design and participants

**Fig. 1:**
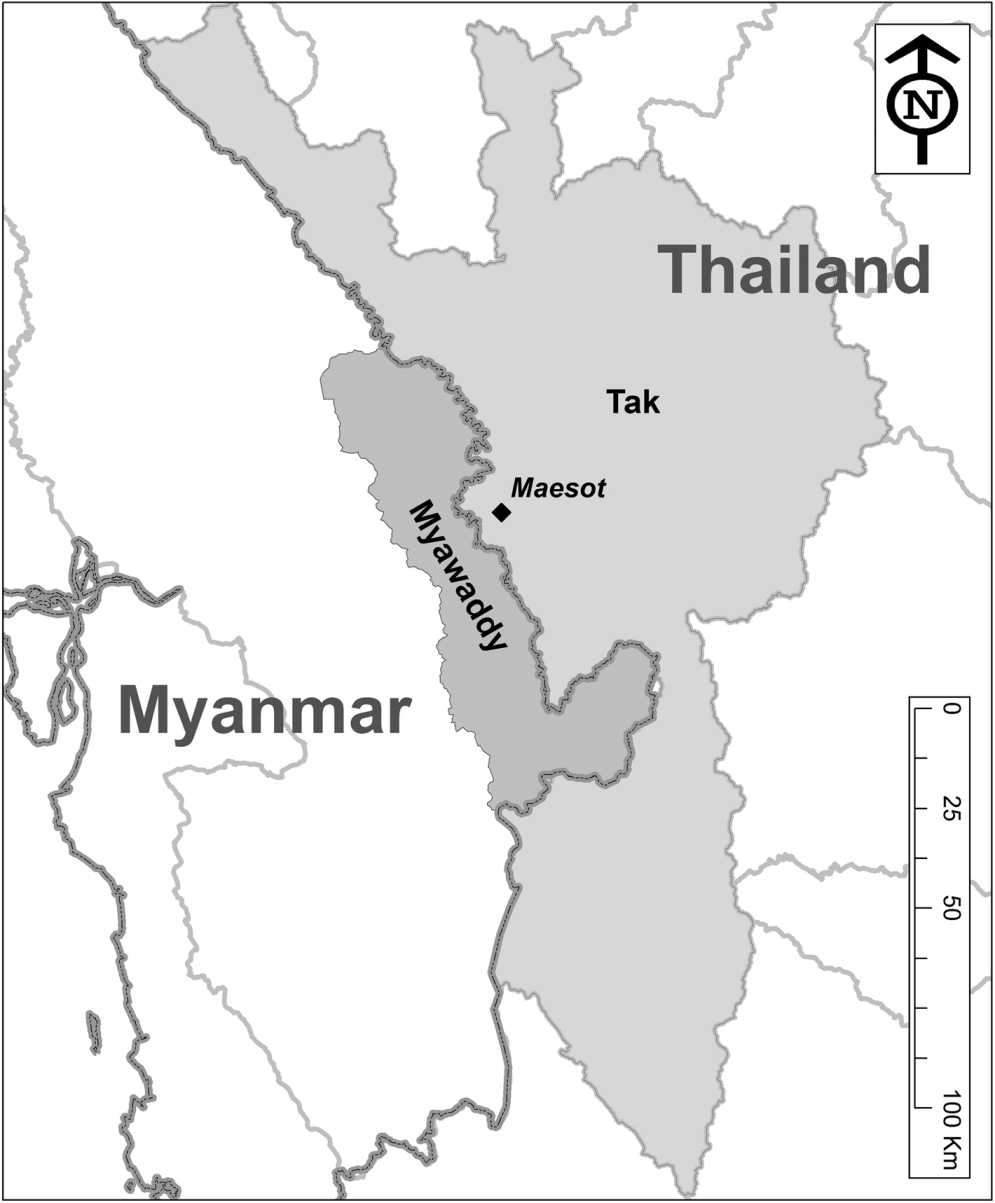
Map of Tak Province, Thailand and Myawaddy District, Kayin State, Myanmar

The study was conducted in Tak province, Thailand and Myawaddy district, Kayin state, Myanmar from November 2013 to July 2014 (Fig. 1). The study team used a qualitative study design with semi-structured in-depth interviews. Purposive sampling was used for this study. One or two key persons from each governmental and non-governmental agency involved in TB control in the two border areas were approached to participate in the study. Some organizations did not have more than three persons responsible for TB control. The interviewees included health policy makers and health care providers involved in TB control at policy development and dissemination, and service provision levels in both countries. In each facility, the most senior staff was selected since they were considered to have the most experience in TB control and care. The interviewees were informed by an introductory e-mail or letter with a participant information sheet and consent form and were given the written sheet and consent form for the participation in this project on site. The sample size for the study followed the concept of saturation since there is no exact way of determining sample size in qualitative research [13].

The study team consisted of a public health officer and medical doctors who have worked along the Thai-Myanmar border for years. One of the medical doctors specializes in TB medicine. The public health officer conducted the interviews on site and took field notes. The two interpreters for interviews conducted in Thai language were native speaking Thais who were fluent in English. The two interpreters for interviews conducted in Myanmar were native-speaking Thais who were also fluent in Myanmar and English.

### Data collection

Data collection was three-fold: (i) collection of baseline information such as TB policies, (ii) conduction of semi-structured in-depth interviews; and (iii) observations made at the health care organizations. The study team gathered baseline information to identify TB policies and programs available on websites, brochures, and publications before interviews. The semi-structured in-depth interviews were the main method of data collection. The interviews were conducted either in English, Myanmar or Thai. The interviews explored existing relationships and interactions across health care organizations and their responsibilities in TB control in the country and across the border. The interview lasted for about 1 h. Interviews were tape-recorded after the participant gave written informed consent.

After an on-site interview, the audio recording was transcribed in a computer. Observations were also made at health care organizations on the day the study team conducted an interview with the stakeholder. Observations included field descriptions of activities, behaviors, and activities of health care personal at TB clinics. Field notes were collected through these observations, interviews and written documents which the study participants gave the study team.

### Data analysis

Data was analyzed through an inductive approach. Data transcriptions were coded and concurrently analyzed using NVivo software (Version 10) for qualitative data analysis. Analysis focused on reviewing segments with similar codes and examining relationships among different codes. Coding also developed categories into a model which summarized the raw data and lead key themes. Two persons independently read and analyzed the same set of transcripts and then compared the results. The analysis reached good concordance between the two researchers and did not show disagreement.

The interviews, field notes and baseline information were utilized to develop a conceptual framework on stakeholders in TB control in the two bridging jurisdictions.

### Ethical considerations

Ethical approval for the study was obtained from the Oxford Tropical Research Ethics Committee in the UK. The approval covers components regarding participants in both Thailand and Myanmar. The study team sought advice from the Tak Province Border Community Ethics Advisory Board (T-CAB) regarding ethical approval in Thailand as well as Myanmar. The T-CAB board members consisted of non-governmental organizations (NGO) workers, teachers and villagers who live in either Thailand or Myanmar [14]. The study team was informed that official approval was not needed since the primary aim of the study was to explore perspectives of stakeholders in TB control and the study subjects did not include vulnerable populations such as patients with TB, refugees and undocumented migrants. The study team received verbal permission from the Tak provincial public health office in Thailand and the Department of Health in Kayin State, Myanmar. The interviewees were informed by an introductory e-mail with an electronic participant information sheet and consent form and were given the written consent form on site. All the consenting key persons were included in the study. Privacy and confidentiality for the interview was ensured through anonymized data. Each interview was identified by a number instead of the name of interviewees.

## Results

Table 1 presents the number of participants and agency types. An introductory e-mail for participation in the study was sent to 36 health care professionals. Among them, 31 respondents agreed to participate in the study; two health care personnel did not respond; two officers reported their organizations were no longer involved in TB control on the Thai-Myanmar border; one officer was willing to participate, however could not make an appointment during the study period.

**Table 1:** Study participant characteristics

	Study participants (N)	
Agency type	Thailand	Myanmar
Government at local and national levels	3	1
United Nations	2	1
Public hospital	5	2
Non-governmental organization (NGO)	6	3
Community-based organization (CBO)	2	6
Total	18	13

Of the total 31 interviews, 21 were conducted in English. Five interviews were conducted in Thai and five conducted in Myanmar. These interviews were then transcribed and translated to English for analysis.

### Conceptual framework

**Fig. 2:**
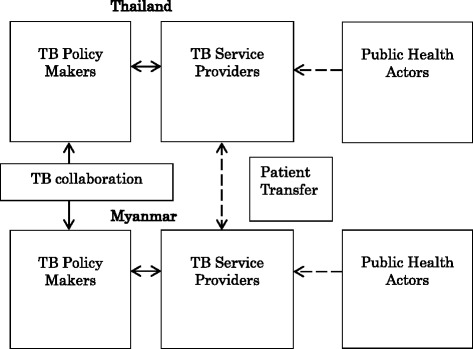
Conceptual framework on stakeholders in TB control

Figure 2 shows a conceptual framework on health care organizations in TB control on the Thai-Myanmar border. The framework was developed through analysis of documents and information obtained from study participants. The TB policy makers such as the national TB program in both countries have the partnership supported by the World Health Organization [15, 16]. They disseminate new policies and plans regarding TB to public health offices at the regional level [9, 15]. The public health offices transmit the policy to TB service providers such as public hospitals and international NGO at the local level. The service providers implement TB programs including diagnosis and treatment services for TB and also receive presumptive TB patients from public health actors such as community-based organizations (CBO) [9, 15].

**Fig. 3:**
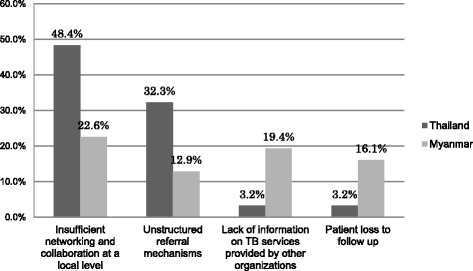
Proportion of the challenges stratified by country (%)

The study identified 19 major challenges and they were categorized into five themes (Table 2). The study revealed limited corroboration and coordination among health care organizations were most frequently reported which led to the main theme in the study. Figure 3 presents the proportions of the challenges in the main them stratified by country. The proportions were calculated by dividing the number of respondents who reported the challenges by the total number of respondents in each country.

**Table 2:** Challenges to TB control on the Thai-Myanmar border based on perspectives of informants

Key theme	Category	Number of respondents mentioning the key theme	Percentage
Coordination and collaboration among stakeholders	Insufficient networking and collaboration at a local level	22	71.0 %
	Unstructured referral mechanisms		
	Lack of information on TB services provided by other organizations		
	Patient loss to follow up		
Service delivery	Inadequate access to TB services	12	38.7 %
	Transportation problems		
	Uncertain sustainability of TB programs implemented by NGOs		
	Impractical policy at a local level		
Organization structure	Shortage of human resources	11	35.5 %
	Limited staff capacities		
	Limited funding for non-Thai patients		
	High workload in non-Thai TB cases		
Epidemiological trend and case finding	High default rate among non-Thais	7	22.6 %
	High MDR-TB case rate among non-Thais		
	Need for effective case finding		
Socioeconomic status of patients	Delay and interruption in seeking care	7	22.6 %
	Limited knowledge and understanding of TB		
	Patient legal status		
	Language barriers

### Coordination and collaboration among stakeholders

Health care providers reported challenges on insufficient networking and collaboration among organizations within country and across the border. The majority of health care providers at the local level stated they did not have regular communication among themselves. Insufficient communication and limited information on TB service provision by other organizations prevented health care providers from engaging in the TB control efforts in the border areas (Fig. 3).

Health care providers raised concern about patients lost to follow up due to high mobility, incorrect home address of patients, unstructured information sharing and lack of patient tracking within country. In particular, many informants in Myanmar expressed concern about patients lost to follow up (Fig. 3). In order to trace patients, some health care providers used official transfer forms written in English which were developed by the National TB program of Thailand and Myanmar. In contrast, other informants did not know the existence of these transfer forms. Once the form reached another organization, part of the form was designed to be sent back to the transferring organization. Although the transferring organization used the form, two health care organizations reported they did not receive their section of the form from the receiving organization.

“*We need to move in a practical relationship like if I want to refer (patients), I have to call this person and fill this form. Always, we have a lot of strategy committees here, but nothing practically.*” (Medical coordinator, NGO)

An informant who received the transfer form with a TB patient from a cross border facility responded that there are difficulties in returning the form due to unreliable postal services, costs, and work burden. Many informants emphasized the need for strengthening the referral system and feedback system by means of a phone call, e-mail and regular meetings. In addition, the study identified misunderstandings among informants in both countries. Whereas interviewees from one side believed that they could not transfer patients who did not have a household registration number, interviewees on the other side said they could treat patients regardless of the household registration number.

During this study period, a cross-border TB/HIV task force meeting was organized by relevant agencies across the border. Public hospitals in Tak and Myawaddy also entered into a memorandum of agreement for service provision.

### Service delivery

Over one third of respondents reported that there are constraints in accessing TB diagnosis and treatment services in hard-to-reach areas due to the terrain and limited transportations. Although there is a high demand for MDR-TB treatment services in border areas, the informants raised concerns about insufficient health care settings that provide comprehensive TB services. International NGOs play an integral role in the provision of TB services to undocumented migrants on both sides of the border. Although acknowledging their importance, interviewees from public hospitals expressed concern regarding the sustainability of TB programs implemented by NGO since NGOs’ programs depend upon external funding.

“*If TB programs of NGO are stopped, they have to hand over the remaining patients to us…If any NGO stops their TB programs, it will be our workloads.*” (Medical doctor, Public hospital)

Health care providers and policy makers mentioned impractical policies at the local level. The informants said that an implementation plan is not often written in detail in the national strategic plan. The national TB program is broad and does not specify practical strategies at the local level. It is not easy for local service providers to contextualize the national policy in the actual delivery of services. There is a need to perform a local assessment of partnerships and roles among stakeholders in order to best accomplish the goals of the national plan. Some respondents suggested developing objectives and implementation plans among relevant organizations at the local level.

### Organization structure

Many organizations faced challenges in shortages of human resources and limited staff capacities. In fact, one CBO terminated their TB program in 2014 due to manpower issues. High turnover rate, high workloads and multitasking for staff serving in TB programs were major issues within the organizations interviewed.

*“We have only one or two TB coordinators who look after 8 – 9 provinces. It is not enough at the regional level. Even in the provinces, we have one who looks after communicable diseases, not only TB, but other infectious disease as well. Those communicable diseases are dengue fever, HIV/AIDS, and Malaria. You can imagine how they can cover surveillance systems and send the report to the central level. It is hard for them.”*(Public health officer, Government sector)

### Epidemiological trend and case finding

Health care providers expressed concern about undetected TB cases, increasing numbers of loss to follow up and MDR-TB cases among non-Thai patients, particularly undocumented migrant patients. Undocumented migrant patients who seek medical care often interrupted treatment, a known factor leading to MDR-TB.

“*It is just like ice berg phenomena. We can see only the patients who come to the clinic. We can see only few patients. There might be a lot of migrant people who may suffer from TB. We couldn’t get them.*” (Medical doctor, NGO)

### Socioeconomic status of patients

Several informants described migrant patients who had advanced symptoms when they reached health care services. Language barriers, legal status, fear of losing employment and arrest, and incarceration or deportation on the way to health care settings prevent patients from accessing health care services early. In addition, some patients have difficulties in understanding the nature of infection with tuberculosis which further hinders patients from adhering to their treatment once they are diagnosed and treatment is initiated.

*“Migrants, they have very challenging socioeconomic situations. They come to us only when they are unable to work.”*(Medical doctor, NGO)

## Discussion

The main theme reported by participants was the challenge of limited corroboration and coordination among stakeholders. Other themes followed to the main key challenge. Addressing the main challenge requires efforts at all three levels: local, national, and bi-national. Many of these challenges can be effectively addressed by empowering local agencies to implement action plans that appropriately adapt national policies to actual conditions. This would create more stakeholder engagement in case finding and treatment and better continuity of care. Thus, the cross-border TB/HIV task force and bilateral agreement between public hospitals are expected to improve referral mechanisms at the local level. The results of this study reflect findings of other studies done in areas where migration issues impact TB control. Recommendations made by a consultation meeting on tuberculosis and migration organized by the World Health Organization Regional Office in Philippines and a study in the Democratic Republic of the Congo have previously highlighted that cross-border coordination and referral of TB patients can be improved by establishing links among relevant stakeholders in border areas where human mobility is high and TB is endemic [17, 18]. Therefore, it will be important to formalize a partnership for consultation and dialogue among key informants on the Thai-Myanmar border. In the dialogue, current referral mechanisms can be reviewed and harmonized within the local context. The multi-stakeholder partnership is expected to conduct stakeholder analysis and develop practical implementation plans among relevant organization at the local level.

The study was conducted in an era of transition for both countries. Political reforms in Myanmar have created new opportunities for international assistance to be deployed within the country and have created a more open collaborative approach to problem solving with its neighbors. Political transition in Myanmar is also generating discussions of repatriation of Myanmar refugees in Thailand [19]. For Thailand, migrant labor remains an ever more important component of the economy [6]. The initiation of the ASEAN Economic Community which goes into effect in 2015 will allow for free movement of goods, services, skilled labor and a freer flow of capital [20]. This regional transformation will inevitably lead to increased movements of people across borders in ASEAN countries, making control of communicable diseases such as TB a regional issue [9]. Thus, case detection of TB will be more challenging in this region. Although health care providers raised concern about passive case finding in our study, active case finding requires financial resources, time, laboratory capacity and supply chain [21]. Mass media campaigns for raising awareness, community engagement and strong partnerships with employers of migrant workers may address the issue [9, 21, 22].

In order to improve service delivery, development of a patient-centered approach such as family member DOTS or community-based models need to be considered with strong counseling and social protection for migrants [12]. Provision of transportation and food, and income generation during treatment may be successful interventions for improving TB control in the vulnerable population.

Removing the financial barriers to treatment will be a key factor in improving TB treatment [23]. Recently, Thailand has introduced a new insurance scheme for migrant workers. The scheme aims to provide universal health care including TB services to all migrants not covered by the Social Security Scheme [24]. Utilization of this approach that aims to cover both documented and undocumented migrants will be watched closely for its acceptability and effectiveness. The scheme could contribute to mitigating barriers to health care among migrant patients and result in more effective finding of new TB cases. Other factors that would have an impact include coordination between health officials and law enforcement to improve access for undocumented migrants who are seeking treatment for tuberculosis.

As respondents indicated, their organization structure makes it difficult to engage in TB control. The problem related of organization structures and implementation of TB programs has also been highlighted in past studies [25–27]. An adequate health workforce is an essential component to achieving improved TB control. In Myanmar, development of human resources and capacity building are urgent since the prolonged conflict devastated health care systems which led to lack of human resources in the border area [28].

Our study has limitations. The sample is limited in terms of the setting and number of participants and may not be representative. Second, the research team conducted interviews in English. Ten interviews were conducted in Thai or Myanmar languages and translated to English which might have caused misinterpretation in the process of the interview and transcription. Third, the study team belongs to a NGO that provides diagnosis and treatment of TB to migrants in Tak province, Thailand and collaborates with other organizations. Potential interviewer bias and respondent bias need to be considered.

## Conclusion

This study explores the practical challenges in the control of TB faced by the stakeholders from various sectors at policy and service provision levels. The findings suggest a general agreement that collaboration among stakeholders in the border areas is insufficient and should be improved to combat TB more effectively. The interviewees also expressed a broad willingness to work together to improve bi-national collaborative mechanisms. Additional support and resources from governmental and non-governmental agencies will be required in the near future to overcome these challenges. These findings may contribute to developing a practical road map for collaborative TB control on the Thai-Myanmar border. It is also expected that these findings may give a new insight to TB control in the similar settings of Southeast Asia.

## Abbreviations

TB: Tuberculosis
MDR-TB: Multidrug-resistant TB
DOTS: Directly observed treatment short-course
T-CAB: Tak Province Border Community Ethics Advisory Board
NGO: Non-governmental organization
CBO: Community-based organization
